# Fabrication of a Highly NO_2_-Sensitive Gas Sensor Based on a Defective ZnO Nanofilm and Using Electron Beam Lithography

**DOI:** 10.3390/mi14101908

**Published:** 2023-10-06

**Authors:** Zhifu Feng, Damiano Giubertoni, Alessandro Cian, Matteo Valt, Matteo Ardit, Andrea Pedrielli, Lia Vanzetti, Barbara Fabbri, Vincenzo Guidi, Andrea Gaiardo

**Affiliations:** 1 Istituto Italiano di Tecnologia, Via Morego, 30, 16163 Genova, Italy; 2 Micro-Nano Characterization and Fabrication Facility Unit, Sensors and Devices Center, Bruno Kessler Foundation, Via Sommarive 18, 38123 Trento, Italy; giuberto@fbk.eu (D.G.); acian@fbk.eu (A.C.); mvalt@fbk.eu (M.V.); pedrielli@fbk.eu (A.P.); vanzetti@fbk.eu (L.V.); 3 Department of Physics and Earth Science, University of Ferrara, Via Saragat 1, 44122 Ferrara, Italy; matteo.ardit@unife.it (M.A.); barbara.fabbri@unife.it (B.F.); vincenzo.guidi@unife.it (V.G.)

**Keywords:** gas sensor, ZnO, MEMS, electron beam lithography, low power consumption, nanofilm, oxygen vacancies

## Abstract

Hazardous substances produced by anthropic activities threaten human health and the green environment. Gas sensors, especially those based on metal oxides, are widely used to monitor toxic gases with low cost and efficient performance. In this study, electron beam lithography with two-step exposure was used to minimize the geometries of the gas sensor hotplate to a submicron size in order to reduce the power consumption, reaching 100 °C with 0.09 W. The sensing capabilities of the ZnO nanofilm against NO_2_ were optimized by introducing an enrichment of oxygen vacancies through N_2_ calcination at 650 °C. The presence of oxygen vacancies was proven using EDX and XPS. It was found that oxygen vacancies did not significantly change the crystallographic structure of ZnO, but they significantly improved the electrical conductivity and sensing behaviors of ZnO film toward 5 ppm of dry air.

## 1. Introduction

Air pollution is a major threat to the natural environment and sustainable social development. Gas analysis can provide key information about the environmental status, which contributes to monitoring the air quality and controlling pollutants [1,2]. Due to the rapid progress of civilization, harmful exhaust gases, including nitrogen compounds, carbides, sulfides, and volatile organic compounds, are emitted in large quantities every day, with a negative impact on human health [3]. Among these gases, NO_2_ is one of the five main air pollutants (together with CO, O_3_, SO_2_, and dusts) causing respiratory diseases, such as bronchitis, pulmonary edema, and asthma [4]. Therefore, NO_2_ detection can be extremely crucial for health preservation and environmental protection.

Nowadays, gas sensors are essential for specific applications such as indoor gas poisoning detection, factory gas explosion monitoring, the drunk driving (exhaled gas) test, and automotive exhaust analysis [5,6,7]. Chemoresistive gas sensors, especially metal oxide semiconductor (MOS)-based gas sensors, have been widely studied and developed due to their remarkable gas-sensing performance and low cost compared to analytical instruments [8]. SnO_2_, ZnO, TiO_2_, In_2_O_3_, and WO_3_ are the most common MOS sensing materials that have been investigated and used [9,10,11]. Among them, ZnO, an n-type semiconductor, is broadly used for gas-sensing applications due to its multiple advantages such as wide direct bandgap (3.2 eV) at room temperature, easy synthesis, non-toxicity, and low production cost [12]. Furthermore, research activities to investigate doping, compositing, morphology modification, and heterostructure fabrication are proceeding to improve ZnO gas sensor performance [13,14,15]. In addition, oxygen vacancy formation is a simple method to improve the ZnO sensing ability [16,17,18,19]. It is well known that oxygen plays a significant role in the sensing process, which is based on the working mechanism of oxygen ionosorption and a modulation of the amount of surface adsorbed oxygen, which determines the electronic properties of the metal oxide sensing materials [20]. In fact, the oxygen vacancies can attract electrons from the valence band, thereby increasing the electron concentration and boosting the reduction of adsorbed O_2_ to oxygen ions. Li and coworkers have found that the oxygen vacancies created by post-annealing can promote the adsorption of oxidizing target gases such as NO_2_ and lead to a dramatic increase in their response [21]. It has also been reported that bulk oxygen vacancies can shift the absorption wavelength from the ultraviolet to the visible region and reduce the bandgap energy due to the formation of traps between the valence and conduction bands [22,23,24,25,26]. The decrease in the bandgap energy also enables a decrease in the operating temperature of metal oxide gas sensors, reducing the power consumption of the MOS sensor, which is typically high for stoichiometric ZnO (>350 °C, depending on the target gas to be detected), and thus reducing the device power consumption [27,28]. To generate oxygen vacancies in MOS, several methods have been studied, such as doping, reduction, and thermal treatment [29,30,31]. 

With the rapid emergence of the Internet of Things (IoT) and the development of machine learning, the newfound usefulness of collected physical and chemical data promotes the application of various gas sensors in multiple locations, which calls for portable gas sensors with high integration and low power consumption. Miniaturization of gas-sensing devices is one of the most efficient ways to reduce power consumption and improve integration with other electronics. Moreover, geometry reduction leads to a low cost, fast response, high sensitivity, and uniformity of temperature distribution over the hotplate [27,32]. Silicon-based MEMS technologies are widely used to fabricate miniaturized semiconductor gas sensors.

For instance, Wang and coworkers fabricated interdigitated gold electrodes and heaters on Si/Si_3_N_4_ substrates using a lithographic technique and electron beam evaporation, and deposited 20 nm SnO_2_:NiO sensing material through magnetron sputtering [33]. The throughput of photolithography is a unique advantage, but the resolution is generally above 1 μm scale, which is short for sub-micro/nanoresearch prototype design. FIB is a direct writing technique that can achieve nanoscale-resolution products. For example, a 25 nm diameter antimony nanowire for a gas sensor was fabricated by using a focused Ga ion beam to detect ethanol and H_2_O with high selectivity [34]. However, it is a very time-consuming way to fabricate micro/nanosized gas sensor device arrays. Electron beam lithography (EBL) is another versatile maskless tool for scientific prototype layout patterning or combining other lithography processes with high precision of electron-sensitive resists [35]. Meanwhile, electron beam scattering (back scattering and forward scattering) in the photoresist and substrate will cause a proximity effect and damage the layout pattern resolution, especially for dense layouts [35]. This occurs since the parameters of EBL, such as accelerating voltage and electron dose, have a great influence on the layout exposure results, and it might be difficult to define patterns containing micro- and nanoparts in the same structures in terms of the time and pattern resolution. If the electron beam dose is too high, overexposure may occur, resulting in round corners and merged gaps. On the contrary, a low electron beam dose will cause underexposure, and the designed patterns will not make contact well. In addition, the electron accelerating voltage and proximity effect adjustment are quite critical for exposure of different sizes and structural layouts. To solve this issue, a method was proposed to fabricate microstructures by combining ultraviolet nanoimprint lithography (UV-NIL) and EBL techniques. The microscale patterns were first imprinted using NIL, and then the sub-microcapillary flow path structure was defined using EBL, which was an efficient approach to fabricate the complex microfluidic devices and is very useful for lab-on-a-chip applications requiring fine and complicated flow paths [36]. However, the method of combining machines requires multi-step processes and marker–pattern alignment, which takes too many resources and too much time. To reduce the proximity effect during EBL exposure, proximity effect correction (PEC) methods, such as incident electron beam dose correction or layout shape correction, have been studied [37,38,39]. Nevertheless, the patterns are generally multifarious, and under some conditions, the proximity correction does not work very well [39].

In this work, a two-step EBL nanofabrication approach was first used to develop a defective ZnO-based chemoresistive gas sensor. This two-step exposure has been developed to balance the proximity effect and the time-consuming patterning of the nanoscale layout inlaid with the dense sub-nanoscale structure of the hotplate (SMHP). It has been developed for MOS gas sensor applications, to decrease the power consumption compared to a bulky micro-level hotplate. Low acceleration was used to expose the micrometer-scale areas of the layout in a large writing field, providing a short exposure time, and a high accelerating voltage was used to expose the dense sub-micro heater circuit in a small writing field, resulting in a vertical and neat sidewall. Then, a sputtered ZnO nanofilm was selected as the sensing material and treated at a high temperature in a pure N_2_ environment to create oxygen vacancies, showing a high response to NO_2_. The ZnO film was characterized in terms of topography and crystallinity, while the sensing ability of the devices for NO_2_ gas was tested under different conditions.

## 2. Materials and Methods

### 2.1. Fabrication Process

**Figure 1:**
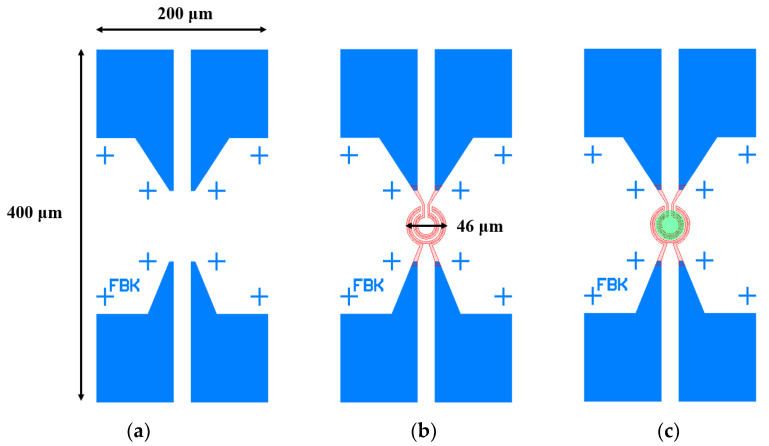
ZnO gas sensor layout patterning steps: (**a**) first step, fabrication of SMHP pads; (**b**) second step, fabrication of heater circuit; (**c**) exposure of active area for sensing material ZnO deposition.

**Figure 2:**
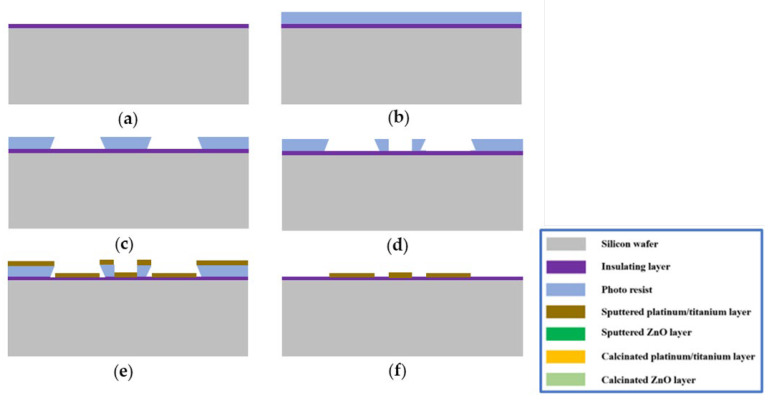
Hotplate fabrication process: (**a**) preparation of wafer with top insulating stack structure; (**b**) spin-coating photoresist on the wafer; (**c**) first step, EBL exposure on the electrode part; (**d**) second step, EBL exposure on the heater circuit; (**e**) metal Pt/Ti sputtering deposition; (**f**) lift-off process.

The exposure process of SMHP is shown in Figure 1, comprising the micro part of the SMHP exposure (Figure 1a); the heater circuit part of the SMHP pattern (Figure 1b), and the definition of the sensing material area (Figure 1c). The details are as follows:

A single-sided polished p-type 600 μm silicon wafer with crystal orientation <100> and a resistivity of 10–20 Ω·cm was selected as the substrate wafer. In the preparation steps, the insulating stack structure including SiO_2_/SiN/SiO_2_ layers was deposited on the top of the silicon wafer, as in reference [40]. Then, the photoresist (PMMA) was spin-coated on the above-prepared wafer with a thickness of about 500 nm. The device pads were patterned using an electron beam with an energy of 10 keV and dose of 250 μC/cm^2^, as shown in Figure 1a. Subsequently, the substrate was developed and a second EBL process was performed (Tescan Mira system equipped with a pattern generator): aligning with the markers was possible to complete the design with the heater circuit using a 30 keV electron beam and dose of 300 μC/cm^2^, as shown in Figure 1b. Metal titanium (Ti) and platinum (Pt) were deposited through electron beam evaporation (ulvac EBX-16C with Ferrotec EV S-6 e-gun from ULVAC technologies) successively, with thicknesses of 10 nm and 100 nm, separately. The lift-off process was implemented in acetone at 40 °C. The whole fabrication process is illustrated in Figure 2.

**Figure 3:**
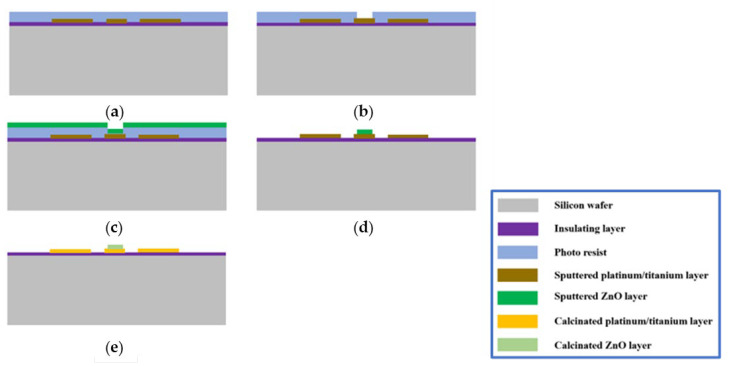
ZnO sensing material deposition: (**a**) photoresist spin-coating; (**b**) ZnO deposition area; (**c**) ZnO nanofilm sputtering; (**d**) lift-off; (**e**) calcination.

The process of ZnO nanosensing film deposition on the hotplate was carried out based on the EBL lithography and lift-off process (Figure 3). The details are as follows: the photoresist was spin-coated on the above-prepared hotplate, and then the ZnO deposition area was defined by using EBL with an energy of 30 keV and dose of 300 μC/cm^2^. A 100 nm thin film of ZnO was deposited using magnetron sputtering (Kenosistec KS 800 C). After lift-off, the final device with the ZnO thin film was calcined at 650 °C in a pure N_2_ atmosphere for 2 h, using an Expertech CTR 200 furnace.

### 2.2. Characterization Tools

The morphology of the samples was investigated by optical microscopy and scanning electron microscopy (SEM, Thermo Fisher Scientific Helios Dual Beam FIB-SEM). The chemical composition analysis of the samples was analyzed by energy-dispersive X-ray spectroscopy (EDX) using the same instrument. The surface roughness of the ZnO nanofilm was characterized by high resolution noncontact silicon atomic force microscopy (AFM, NT-MDT Spectrum Instruments). The crystal structure was revealed using a Bruker D8 Advance Da Vinci diffractometer operating in Bragg–Brentano geometry with a Cu-anode X-ray tube and a Ni filter to suppress the contribution of the CuKβ component, and a LynxEye XE silicon strip detector (angular range covered by the detector = 2.585° 2θ) calibrated to discriminate CuKα radiation. The samples were placed in a height-adjustable poly (methyl methacrylate) specimen holder and scanned in a continuous mode from 5 to 90° 2θ with a step size of 0.02° 2θ and a counting time of 2 s per step. A Kratos AXIS UltraDLD instrument (Kratos Analytical, Manchester, UK) was used to conduct the XPS characterization. With the emission angle between the analyzer axis and the normal to the sample surface at 0°, the sampling depth is roughly 10 nm. Survey spectra and O 1 s, C 1 s and Zn 2p core levels were acquired for each sample. The C 1 s hydrocarbon peak at 284.7 eV was used to calibrate the XPS spectra binding energy scale. After Shirley background subtraction, the integrated area of the core levels was used to quantify the elemental content ratio [41]. The resistance of the hotplate was measured by a manual probe with a voltage range of 0 to 6 V, using a Karl Suss Manual probing station PM8 (SUSS MicroTec Semiconductor) equipped with an Agilent 4156C Precision Semiconductor Parameter Analyzer with a nominal resolution of 1 fA and 2 μV. The temperature of the SMHP was calculated by using the Pt temperature coefficient of resistance (TCR), which determines the relationship between the heater electrical resistance and temperature [42,43]. 

### 2.3. Gas Sensing Measurement

After the device calcination treatment, the chips were bonded with gold wire to TO-39 package for the gas sensing measurement. A customized gas sensing test system was used to characterize the sensing ability of the ZnO nanofilm at 25 ± 2 °C [10]. The sensors were installed in a sealed gas test chamber, connected to a peripheral pneumatic line consisting of mass flow controllers and gas cylinders containing certified concentrations of target analytes. The operating temperature of the gas sensors was controlled by the input voltage applied to the micro hotplate and obtained by calculating the TCR of the Pt heater. The sensing film resistance change was measured continuously by the custom data acquisition system. Prior to the sensing material measurement, all sensors were stabilized by aging in synthetic dry air (20% O_2_, 80% N_2_) for several hours. By changing the ratio of dry air (carrier) to target gas using mass flow controllers with a total flow rate of 200 sccm, the required concentration of NO_2_ was achieved. The sensing measurement was also carried out at different relative humidity (RH%). A bubbler filled with deionized water was used to provide humidity in the gas test bench. A commercial humidity sensor connected to the pneumatic system was used to measure the exact RH% in the gas chamber in real time. The baseline resistance (R_air_) of the sensing material in the air was defined as the background reference, and the obtained resistance of the sensing material in the target gas was defined as R_gas_. Hence, the change in resistance of the sensing material under the target gas is proportional to the gas sensing response, which was calculated by using the following equation:

R = R_gas_/R_air_ (1)

To compare the effect of oxygen vacancies on ZnO properties and sensitivities, two groups were prepared, one was ZnO nanofilm after sputtering named as ZnO1, and another group sample was ZnO nanofilm after calcination in N_2_ at 650 °C named as ZnO2. 

## 3. Results

### Structural and Morphological Analysis

**Figure 4:**
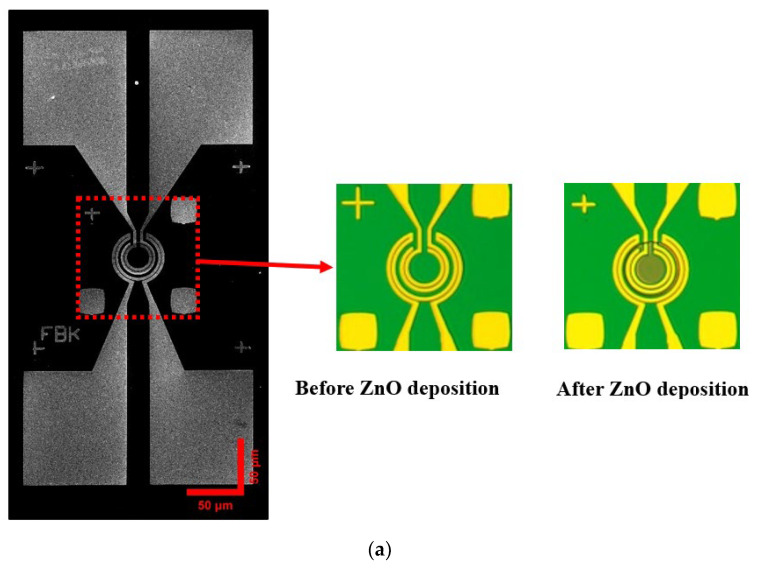

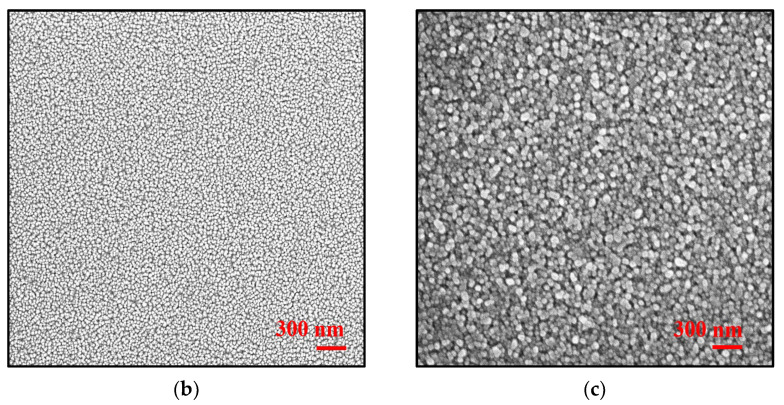
Images of ZnO gas sensor: (**a**) images of ZnO gas sensor structure; (**b**) SEM image of ZnO1 film on SMHP; (**c**) SEM image of ZnO2 film on SMHP.

The final gas sensor SMHP including pad parts and heater part fabricated by the process is illustrated in Figure 4a, which shows the neat structure of hotplate plate and semi- transparent ZnO circular sensing film under optical microscope. The details of ZnO nanofilm were observed as shown in Figure 4b, which shows the homogenous nanoparticles of ZnO with diameter size of about 20–40 nm. After the post-annealing process, the size of ZnO nanoparticles became larger in 60–90 nm, as shown in Figure 4c, proving that the temperature treatment can boost the growth of ZnO nanoparticles. 

**Figure 5:**
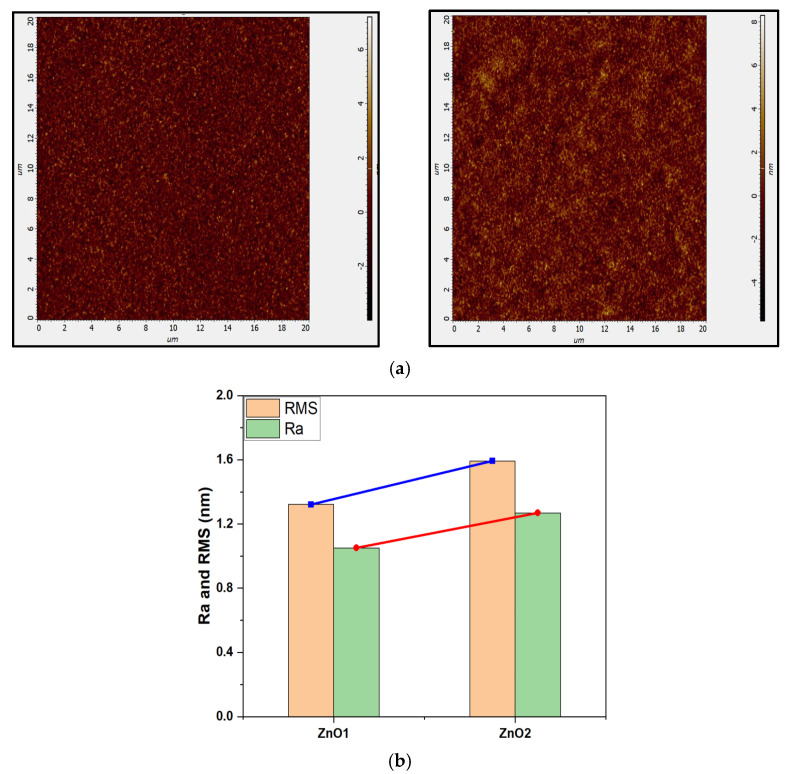
(**a**) AFM images and (**b**) the roughness values of ZnO1 and ZnO2.

The surface morphologies of ZnO1 and ZnO2 films were examined by AFM in Figure 5. The roughness average (Ra) and the root mean square (RMS) of ZnO2 are slightly higher than that of ZnO1, indicating that heat treatment could increase the surface roughness. This may be due to the agglomeration of ZnO particles, which grow into larger granular structures during high temperature heat treatment [44], as evidenced by SEM images.

**Figure 6:**
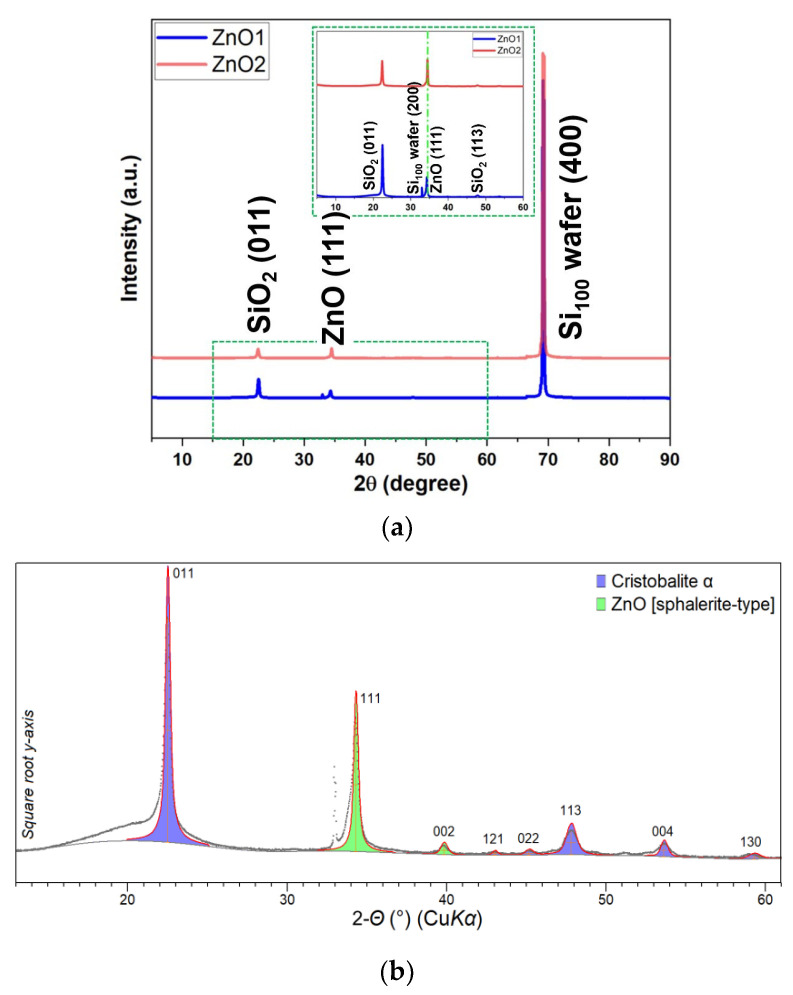
(**a**) XRD patterns of ZnO1 and ZnO2; (**b**) XRD pattern comparison of substrate cristobalite α phase and ZnO.

The XRD powder diffraction patterns for ZnO samples collected at room temperature are shown in Figure 6a. The two XRD patterns show almost identical diffractometric features, indicating that the heat treatment at 650 °C in an N_2_ atmosphere has no apparent effect on the initial phase composition. In addition to the Bragg reflections deriving from the Si wafer used as substrate (i.e., reflection 400 at ~69.2 °2θ, the basis-forbidden reflection 200 at ~33.0 °2θ, the latter due to the multiple diffraction effect as described in [45]), the XRD patterns are characterized by diffraction peaks from other crystalline phases. Namely, a SiO_2_ phase with cristobalite crystal structure with main diffraction peaks centered at ~22.5 and ~47.8 °2θ (corresponding to XRD reflections 011 and 113, respectively), which is probably due to the conversion of the tetraethoxysilane (TEOS) precursor during the synthesis process, and a ZnO phase with a cubic sphalerite-type crystal structure with main diffraction peaks centered at ~34.3 and ~39.8 °2θ (corresponding to XRD reflections (111) and (002), respectively), as shown in Figure 6b [46]. The crystallite size of the ZnO phase, obtained by line profile analysis of the diffraction peaks, is unchanged after thermal treatment and is 29(4) nm.

Table 1 shows the elemental composition in the obtained samples by EDX analysis at ambient conditions. The atoms ratio of O/Zn for ZnO1 is 1.065, which is higher than 1 due to the oxygen in the air. After calcination of ZnO under N_2_ gas at 650 °C for 2 h, the atom ratio of O/Zn decreased, indicating the reduction of oxygen content inside the ZnO crystal structure and the formation of oxygen vacancies. 

**Figure 7:**
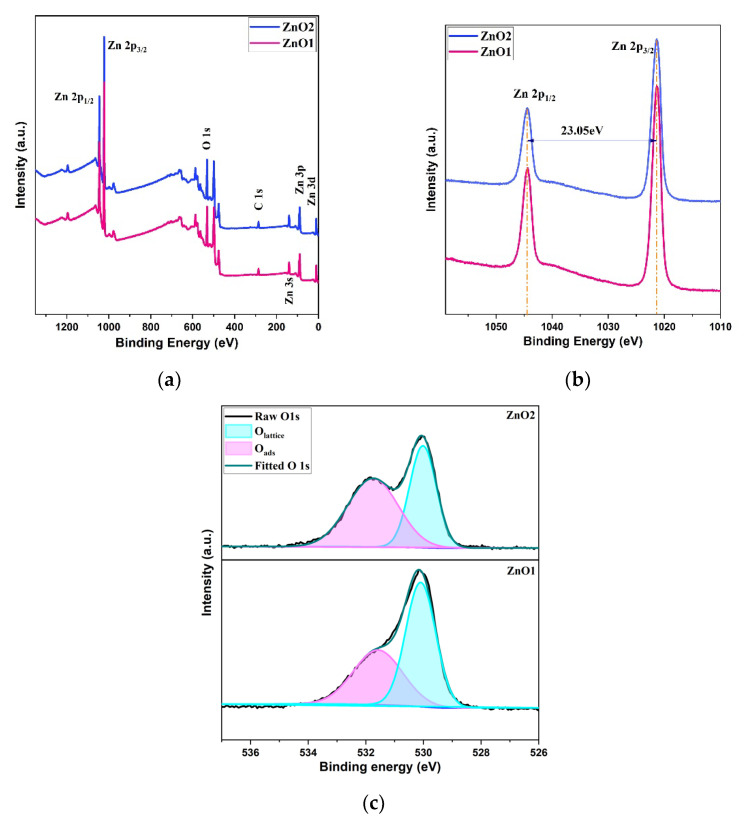
XPS spectra: (**a**) full scan of samples; (**b**) Zn 2p spectrum; (**c**) O 1s splitting peaks of ZnO1 and ZnO2.

XPS measurements were performed by using a conventional monochromatic Al Kα (hν = 1486.6 eV) source and normal emission geometry on deposited ZnO film to analyze the surface elemental compositions and chemical states before and after treatment in N_2_ atmosphere at 650 °C for two hours. The sample spectra are fitted with Gaussian–Lorentzian function and Shirley background. In the survey spectra (Figure 7a), three elements Zn, O and residual C are present in both samples ZnO1 and ZnO2. The residual C is caused by the adsorption from the environment, and the binding energy of 284.7 eV of C 1s hydrocarbon peak is used as a reference in the spectra. The Zn 2p doublet is shown in Figure 7b for both samples. The binding energies of the two peaks are 1021.20 eV and 1044.25 eV suggesting the existence of Zn atoms in the oxidized state. The O 1s core level can be fitted into two components: O_lattice_ corresponding to Zn–O bonding state (EB = 529.97eV) and O_ads_ corresponding to the adsorbed O state (EB = 531.71eV) [47]. Figure 7c shows the fitting of the oxygen core level with two peaks, O_lattice_ and O_ads_ in samples of ZnO1 and ZnO2. The ratio of O_lattice_ to O_ads_ are 1.13 and 0.74, respectively, obtained by integral area ratio calculation, which indicates the decrease in the amount of O_lattice_ within the ZnO crystal structure after calcination in N_2_ environment.

**Figure 8:**
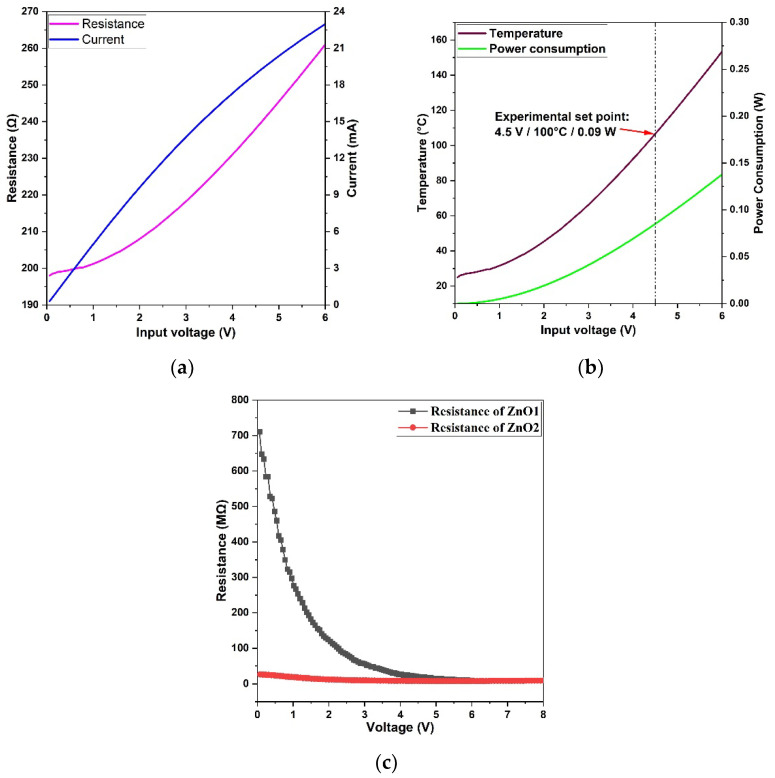
The voltage-dependent performance of the (**a**) current and resistance of hotplate, (**b**) working temperature and power consumption of hotplate, (**c**) resistance change in ZnO1 and ZnO2.

Figure 8 shows the electrical performances of the hotplate and the ZnO sensing film. The current and the corresponding resistance of the hotplate were measured at different input voltages. Because of the heat dissipation and the highly temperature-dependent resistivity of the Pt circuit, the current and resistance of the Pt heater do not have a linear relationship with the input voltage. Accordingly, the temperature and power consumption of the hotplate at different input voltages also show a nonlinear trend. The operational temperature for the ZnO gas sensor was selected as 100 °C at 4.5 V with a power consumption of 0.09 W, at which it showed the optimized sensing performance. The resistance of the ZnO nanofilm was measured at different voltages; by increasing the voltage on ZnO, the temperature of ZnO also increased, which indicated the resistance change in ZnO at different temperatures. Figure 8c shows the resistance change in ZnO1 and ZnO2 at different voltages. The calcined ZnO2 film shows relatively stable resistance values compared to those of the sputtered ZnO1 film at different voltages, which proves that O vacancies can significantly affect the electrical conduction of the ZnO film.

**Figure 9:**
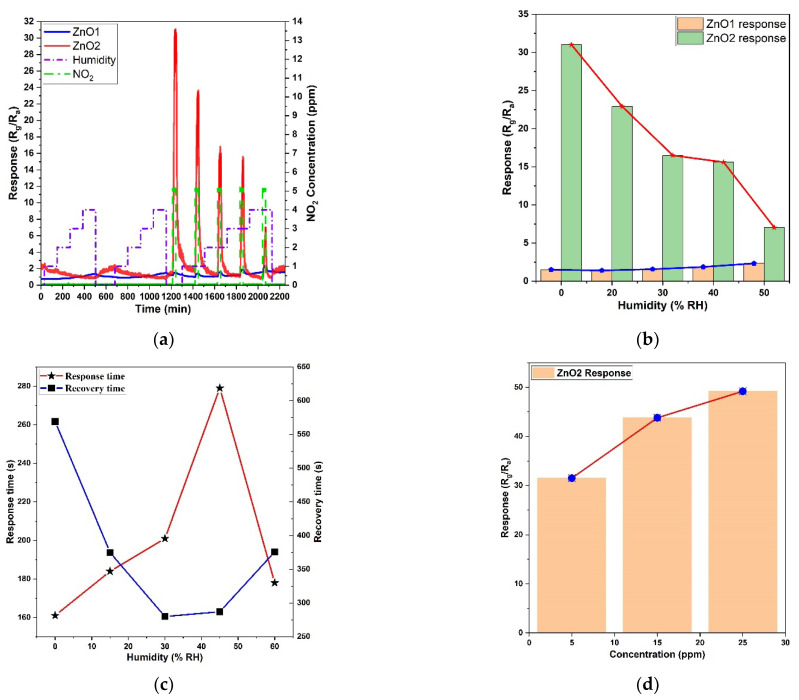
(**a**) ZnO sensing curves toward 5 ppm NO_2_ gas at different conditions; (**b**) ZnO sensing trend toward 5 ppm NO_2_ under different humidities; (**c**) ZnO2 sensing response/recovery time toward 5 ppm NO_2_ under different humidities; (**d**) ZnO2 sensing response toward NO_2_ with different concentrations in dry air.

The sensing ability of the samples was tested toward 5 ppm NO_2_ gas under different relative humidity (RH%) conditions at the beginning. This was a suitable test because humidity is ubiquitous in real-world conditions and is a non-negligible factor that can affect the response of gas sensors. In general, the humidity concentration indoors is between 15% and 60% RH. At the beginning of this gas-sensing test, humidity with increasing concentration was injected in the first two steps, and then NO_2_ target gas with 5 ppm concentration was injected under different humidity concentrations, as shown in Figure 9a. During the sensing process, H_2_O molecules may decompose into two groups, H^+^ and OH^−^, after the dissociation of H_2_O at the operating temperature. Presumably, these two groups will react with lattice oxygen or adsorbed oxygen acting as surface donor and acceptor [48]. The ZnO thin film did not show a clear response to humidity. Instead, the resistance of both samples slightly decreased after the injection of increasing humidity, indicating a weak interaction between ZnO nanofilm and humidity, as shown in the first loop test in Figure 9a. The water vapor molecules decrease the adsorption of oxygen through the formation of hydroxyls, which further weakens the sensing response. The water vapor poisoning function is expressed by the following equation [49]:

(2) $$H_{2}O\left(g\right)+2Zn+O_{ad}^{2-}\left(or\;O_{ad}^{-}\right)\;↔\;2\left(Zn-OH\right)+2e^{-}\;(or\;e^{-})$$

When the humidity was 0, ZnO2 showed a very high response to NO_2_, while, after increasing the humidity concentration from 0 to 60 RH%, the response gradually decreased, indicating the hindered adsorption sites of oxygen on the surface, caused by the water adsorption [50]. ZnO1 showed a slight opposite response trend to NO_2_ in increasing the humidity concentration, as depicted in Figure 9b. The difference between ZnO1 and ZnO2 is the oxygen vacancy concentration, which means that oxygen vacancies could also affect the water molecule adsorption and decomposition [51,52]. Figure 9c presents the response/recovery time for 5 ppm NO_2_ under different humidities, calculated as the time needed to reach 90% of the response value and the time needed to recover 90% of the baseline signal, respectively. The response time and recovery time exhibited totally opposite trends, which indicated that humidity affected the sensing process time. When the humidity was relatively low, increasing humidity resulted in a fast response and slow recovery. However, when the humidity was much higher, the response became faster, and the recovery time could be slowed by increasing humidity.

**Figure 10:**
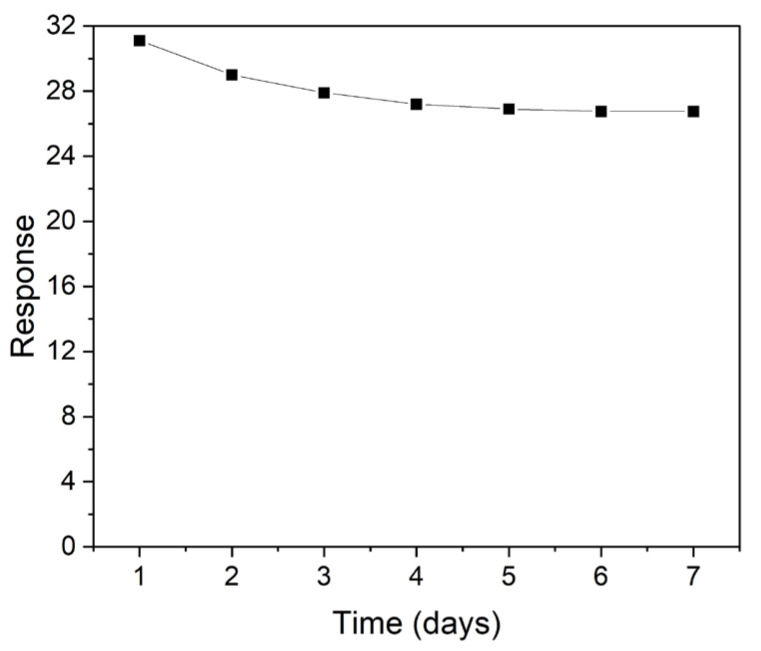
Sensing response repeatability of ZnO2 sensor vs. 5 ppm of NO_2_ over one week of measurements.

The repeatability of the sensor responses over time were also analyzed by exposing the ZnO2 sensor to 5 ppm NO_2_ for seven consecutive days (Figure 10). As can be seen, the sensor showed a substantial drop (around 10%) in the detection response during the first four days, and then it tended to stabilize at a value of 27. 

Table 2 compares the gas sensing performances of nano ZnO materials against NO_2_ gas with some results from the literature. The ZnO2 gas sensor has a super high response to NO_2_ at low concentrations. 

## 4. Discussion

**Figure 11:**
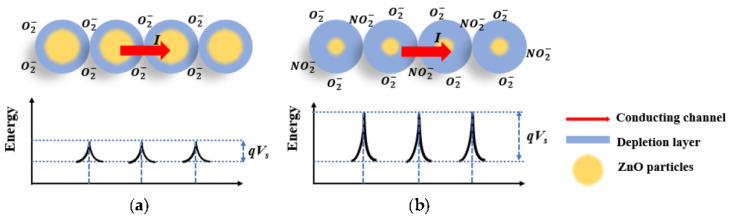
Schematic representation of the conduction mechanisms through ZnO nanoparticles on O_2_ (**a**) and NO_2_ gases (**b**).

The sensing mechanism of ZnO toward NO_2_ is based on two aspects: the receptor function and transducer function. The receptor process mainly involves the oxygen adsorption in the air at a certain operating temperature, which involves the process of electron exchange between the adsorbed gas and the surface of the sensing material. This state is dominated by the surface properties of the sensing material, such as the specific surface area and the electron affinity. The transducer function refers to the transition from the signal caused by the chemical interaction between the adsorbent gases and the sensing material to the detectable electrical signal, which is significantly affected by the grain boundaries of the sensing material. In our case, when the n-type ZnO nanofilms were exposed to the dry air at the operating temperature, oxygen atoms were adsorbed on the surface of the ZnO film, while the electrons in the conduction band of ZnO were attracted by oxygen atoms, resulting in the formation of O^−^ or O^2−^, as depicted in Figure 11a. Since the carrier charges are electrons in n-type ZnO material, the concentration of electrons will be reduced after the adsorption of oxygen. Therefore, the resistance of ZnO will be increased. Once the target gas NO_2_ has been injected around ZnO, the nanofilm will trap more electrons from the conduction band, which will further increase the resistance, as shown in Figure 11b. The ZnO nanofilm did not show any clear response to humidity, but humidity affected the response of the ZnO nanofilm to the NO_2_ target gas, which implied that adsorbed water molecules could occupy the active sites on the surface of ZnO instead of reacting with the O ions on the surface of ZnO. The whole sensing process is shown in Equations (3)–(8) [33,60]:

(3) $$O_{2}\left(gas\right)↔O_{2}\left(ads\right)$$

(4) $$O_{2}\left(ads\right)+e^{-}↔O_{2}^{-}(ads)$$

(5) $$O_{2}^{-}(ads)+e^{-}↔2O^{-}(ads)$$

(6) $${NO}_{2}\left(gas\right)+e^{-}↔NO_{2}^{-}\left(ads\right)$$

(7) $$NO_{2}\left(gas\right)+O_{2}^{-}\left(ads\right)+2e^{-}↔NO_{2}^{-}\left(ads\right)+2O^{-}(ads)$$

(8) $${NO}_{2}\left(gas\right)+O^{-}\left(ads\right)↔NO^{+}\left(ads\right)+2O^{-}(ads)$$

## 5. Conclusions

In summary, the MEMS-based ZnO nanofilm gas sensor was fabricated through two-step EBL exposure, which can define the sub-microstructures on nanopatterns. A low electron accelerated voltage with low electron beam dose was applied to expose the nanopatterns of the hotplate in a large writing field, and a high electron accelerated voltage with high electron beam dose was used to expose the sub-micro heater circuit part in a small writing field. Then, 100 nm thick ZnO thin film was deposited through magnetron sputtering to cover the hotplate, and it was treated in a pure N_2_ environment at 650 °C to create oxygen vacancies, which can modulate the conduction mechanisms and the oxygen adsorption ability of ZnO nanofilm. The gas sensing of ZnO was tested for 5 ppm NO_2_ under different humidity conditions, which proved both that the oxygen vacancies can affect the conductivity of ZnO nanofilm and that there is a super high sensing response of ZnO2 toward NO_2_. ZnO nanofilm did not show a clear response to humidity, but water molecules inhibited the sensitivity to NO_2_. At the same concentration of NO_2_ and the operating temperature, higher humidity caused a lesser response.

The MEMS-based MOS gas sensors are expected to be widely adopted given their advantages of low cost, low power consumption, high miniaturization, and integration. In this work, an achievable approach has been shown to fabricate a sub-micro-sized MOS gas sensor device, which can be regarded as a platform and further developed to improve the gas-sensing capabilities. For example, ZnO nanofilm can be doped using a quantitatively controlled noble metal to enhance its sensitivity and selectivity. Furthermore, a p-n junction between two layers of nanofilms can be created via the MEMS process based on our fabrication routine, with a potentially improved sensing performance compared to a sending film with a single layer. Moreover, a further step, which could be realized based on this work, is to keep miniaturizing the geometrical structure of the gas sensor, working toward much lower power consumption and a high integration concentration. 

## Figures and Tables

**Table 1:** Element ratios of samples obtained from the EDX analysis.

Samples	Zn (at%)	O (at%)	C (at%)	O/Zn (%)
ZnO1	41.29	43.96	14.74	1.065
ZnO2	32.45	33.71	33.83	1.039

**Table 2:** Reported data on nano ZnO material gas sensors’ sensing ability toward NO_2_ from the literature in comparison with those from the present work.

Material	Dimensions	Size (nm)	Operational Temperature (°C)	NO_2_ Concentration (ppm)	Sensor Response (R_gas_/R_air_)	Reference
ZnO nanosheets	2D	80	100	2	2.6	[53]
ZnO	Nanosheets	790	RT	25	23	[54]
ZnO	Nanowire	30–50	RT	1	Around 10	[55]
ZnO	Nanofilm		RT	0.125	5.5	[21]
ZnO	Nanoparticles	100	300	10	6.8	[56]
ZnO	Nanorods	60	100	1	13.4	[57]
ZnO	Nanorods	100	200	1	2.3	[58]
ZnO	Nanorods	50–500	250	10	13	[59]
ZnO	Nanosheets	200	100	1	2.5	[60]
ZnO	Nanoparticles	25–31	100	3%	1.2	[16]
ZnO/SnO_2_	Heterojunction	2000	300	1	1.3	[17]
ZnO	nanofilm	60–90	100	5	31.1	This work

## Data Availability

The data presented in this study are available on request from the corresponding author.
